# Improving communication about HIV prevention among people living with HIV and their at-risk social network members in Dar es Salaam, Tanzania

**DOI:** 10.1080/2331205X.2019.1600230

**Published:** 2019-03-31

**Authors:** Hellen Siril, Anna Kaale, Anna Minja, Japheth Kilewo, Ferdinand Mugusi, Bruno Sunguya, Jim Todd, Sylvia Kaaya, Mary C. Smith Fawzi

**Affiliations:** 1 Department of Psychiatry and Mental Health, Muhimbili University of Health and Allied Sciences, Dar es Salaam, Tanzania.; 2 Department of Public Health Evaluations and Quality Improvement, Management and Development for Health, Dar es Salaam, Tanzania.; 3 Department of Healthy Options, Africa Academy for Public Health (AAPH), Dar es Salaam, Tanzania.; 4 Department of Epidemiology, Muhimbili University of Health and Allied Sciences, Dar es Salaam, Tanzania.; 5 Department of Internal Medicine, Muhimbili University of Health and Allied Sciences, Dar es Salaam, Tanzania.; 6 School of Public Health, Muhimbili University of Health and Allied Sciences, Dar es Salaam, Tanzania.; 7 London School of Hygiene and Tropical Medicine, London, UK.; 8 Department of Global Health and Social Medicine, Harvard Medical School, Boston, MA, USA.

**Keywords:** Social Sciences, Behavioral Sciences, Health and Social Care, HIV, prevention communication, PLH, social network members

## Abstract

Although a number of HIV prevention programs have been implemented, such as mass media campaigns, high rates of unprotected and concurrent sexual partnerships, as well as low uptake HIV testing and limited HIV knowledge, persist in Tanzania. We examined the effect and predicting factors of HIV prevention communication among people living with HIV (PLH) exposed to *NAMWEZA* intervention, and their at-risk social network members (NMs) Quantitative data were collected from 326 participants at baseline and 24 months of follow-up. In-depth interviews with 20 PLH were conducted at follow-up. Results indicated specific communication about condom use and HIV testing increased; (mean increase of 0.28 (SD = 0.14) scores, P = 0.012 and 0.42 (SD = 0.11) scores, p < 0.001 respectively while general discussion about protecting other people from HIV did not change significantly; mean increase was 0.01 scores (SD = 0.005), p = 0.890. Positive predictors of communication included being single; OR = 1.10, p = 0.01, female; OR = 1.15, p = 0.03, aged 30 years or older; OR = 1.23, p < 0.01, HIV knowledge, dose of *NAMWEZA* participation; OR = 1.01, p < 0.001, and high self-efficacy for condom use; OR = 1.4, p < 0.001. Stigma demonstrated a significant but negative association with communication for condom use; OR = 1.01, p < 0.01.Qualitative data reflected perceived possession of more individual skills and ability to address some personal/cultural obstacles to communicating about HIV prevention including those observed in the quantitative data. *NAMWEZA* improved communication about HIV prevention among PLH with their at-risk-NMs. The approach is a promising complement to media campaigns in similar populations. Future research and program evaluation efforts should explore how communities perceive and communicate about protecting others from HIV.

## 1. Introduction

HIV remains a significant public health concern in Tanzania—a country with 1.6 million people living with HIV, 74,000 new HIV infections, and 40,000 HIV/AIDS-related deaths occurring each year. Majority of the new infections are among people between the ages of 15 to 49 years (UNAIDS (2017). Throughout the epidemic, there have been multiple interventions and efforts to address the burden of HIV in Tanzania (Gamell et al., 2017; Karan et al., 2017). Advocating for HIV prevention and educating people on the benefits of seeking and remaining in HIV care are among such efforts implemented across the country, largely through mass media (MoH, 2013).

Despite these efforts, a large proportion of the population has demonstrated a low uptake of HIV prevention approaches, including condom use and HIV testing services. The rate of concurrent sexual relationships, which is more prevalent among men in Tanzania, is 57% yet the country’s 2011/2012 HIV Malaria Indicator Survey (THMIS) report (TACAIDS (2012) revealed that only 15% to 45% of men and 22% to 48% of women used condoms in their sexual encounters. Similarly, 75% of the people surveyed in the THMIS knew where to access HIV testing services but only 47% of men and 62% of women had had an HIV test. The same report indicated that only a small proportion of the population had comprehensive knowledge about HIV: 42% among women and girls and 50% among men. In addition, preliminary results of another survey known as the Tanzania HIV impact Survey (THIS) conducted in 2016/2017 showed that the proportions of people who had received an HIV test and had knowledge of their HIV status were still low and varied widely by risk group—specifically, among men who have sex with men who were HIV-positive only 14% knew their status before they took the test. This is in contrast to female sex workers, among whom 58% knew their HIV status (ICAP (2017). The third national Health Sector HIV/AIDS strategic plan (2013–2017) highlighted that the population of Tanzania still had evidence of social, cultural and economic gaps predisposing some individuals to riskier behaviors that could lead to the acquisition of HIV infection. This plan also emphasized the need to adopt specific evidence-based social/behavior change communication models to advance safer sexual behaviors for HIV prevention (MoH 2013–2017).

Both interpersonal and mass media communication/campaigns are used to provide education in order to increase knowledge and change negative beliefs in order to facilitate HIV prevention (Davey-Rothwell & Latkin, 2007; Duggan, 2006; Zamboni, Crawford, & Williams, 2000). Across the country, due to the large size (945,087 km^2^) and a population of 48 million people mass media education and communication campaigns are mostly used to ensure a broader reach of messages concerning HIV and other disease prevention messages across the population. The most common form of media used is radio, with over 85% of the population in rural areas owning a radio; occasionally television is used but is less common in part due to cost and the smaller proportion of the population that owns a TV in rural areas (Media, 2014)

However, there are additional benefits that interpersonal communication has over media that allow for discussion that might help address specific individual challenges hindering uptake of some of the commonly recommended HIV prevention approaches. Previous studies indicate that lack of practical skills to engage in effective interpersonal communication hinders HIV prevention efforts (Katikiro & Njau, 2012); Talib, Silver, Coupey, and Bauman (2013). Other studies found that interventions to improve interpersonal communication among people living with HIV (PLH) and their At-risk-NMs had greater potential for modeling safer sexual practices and HIV prevention (Arnold, Sterrett-Hong, Jonas, & Pollack, 2016; Noar, Carlyle, & Cole, 2006; Ssali, Wagner, Tumwine, Nannungi, & Green, 2012; Talib et al., 2013; Tobin, Kuramoto, Davey-Rothwell, & Latkin, 2011; Tomori et al., 2014; Zamboni et al., 2000). In general, mass media communications are not complemented with opportunities for interactions and discussions on how the behavior change can be adapted amidst existing challenges, possibly limiting the potential to affect changes. Enhancing interpersonal communication skills for HIV prevention at the community-level can supplement the mass media by adding discussions or interactions that can address individual and communities’ challenges of integrating HIV prevention approaches (Limaye 2013).

In Tanzania, cultural factors still prescribe who can acceptably talk about sex, thus interpersonal communication about HIV preventive communication approaches is limited at the community level (Davey-Rothwell & Latkin, 2007; Kajula, Darling, Kaaya, & De Vries, 2016). While sexual risk behavior is the primary route of HIV transmission in Tanzania, interpersonal communication about sex among parents and children (Mathews et al., 2012; Ross et al., 2007) and among married couples (Exavery et al., 2012; Mtenga, Geubbels, Tanner, Merten, & Pfeiffer, 2016) is limited. Women in Tanzania fear that intimate partner violence may occur if they initiate communication about safe sex with their male sexual partners (McCloskey, Williams, & Larsen, 2005; Sa & Larsen, 2008). Increasing the use of interpersonal HIV prevention communication beyond health facilities into the communities may help address some of these cultural barriers and could positively impact HIV prevention efforts. PLH receiving HIV care and treatment services in health facilities in Tanzania are more likely to have awareness and knowledge of HIV, including prevention approaches, due to their exposure to such knowledge during routine clinic-based counseling sessions. This can provide them with skills for communication that address existing cultural norms, fear, stigma, and negative attitudes towards HIV, which in turn may have an impact on their NMs who are at higher risk of acquiring HIV also called at-risk- NMs and to the larger community (Lettenmaier, Kraft, Raisanen, &, Serlemitsos, 2014). This study sought to assess the effect of the *NAMWEZA* intervention on facilitating HIV prevention communication between PLH with their NMs at risk for HIV infection and to describe experiences of the participants in addressing some of the most common individual and cultural obstacles to HIV prevention communication.

## 2. Methods

### 2.1. Study design, setting, and description of the intervention

A cohort study was conducted between December 2012 and December 2014, examining the predictors of HIV-related communication with NMs among PLH participating in the *NAMWEZA* intervention. It employed a mixed methods approach to data collection and analyses. The quantitative methods availed data to quantify the frequency and predictors of HIV prevention communications and the qualitative data (in-depth interviews) were collected to obtain descriptive experiences of participants on utilizing *NAMWEZA* communication strategies with their at-risk NMs. The study was conducted in two large, urban health facilities providing antiretroviral therapy (ART) services located within the largest district of Kinondoni in Dar es Salaam region, Tanzania.

#### 2.1.1. Description of NAMWEZA intervention

*NAMWEZA* comes from two Swahili language words: *NAM* which means “yes I” and *TUNAWEZA* which means “yes we can” thus the overall meaning of *NAMWEZA* yes I can and yes together we can. It is a manualized psychosocial group training intervention, facilitated by trained peers and offered as 10 once weekly sessions(Siril et al., 2017). The content includes discussions on key psychosocial issues in the lives of PLH and NMs, using components of the Appreciative Inquiry (AI)—a positive psychology model which is a strengths-based approach that involves the art and practice of communicating through “unconditional/none judgmental positive questions” and focusing on communication to build better understanding to facilitate positive relationships and behavior changes (Buck, 2017; Dewar & MacBride, 2017; Issel, 2017; McCarthy, 2017; Moore et al., 2017; Sandars & Murdoch-Eaton, 2017; Teevale & Kaholokula, 2018; Whittaker et al., 2017). *NAMWEZA* removes the element of blaming that fuels anger (Ngure et al., 2016) and hiders communication for HIV prevention. It emphasizes on being positive towards another person and focusing on understanding them and includes mentioning their strengths and not weakness during communication about HIV prevention. In the local context age and gender were some of the main challenges in communicating issues related to sex including HIV transmission in this population. Therefore, we implemented the intervention in four age and gender-specific groups, including younger men (< 40 years of age), younger women (< 35 years of age), older men (≥ 40 years of age), and older women (≥ 35 years of age), comprised of 80–100 participants each with sessions lasting three to four hours. The participants were asked to provide names and contacts of their close social network members that were at high risk of contracting HIV such as female and male sex workers, cohabiting married or unmarried couples, friends or family members with problems with alcohol, among other factors.

### 2.2. Selection of participants and eligibility criteria

We used multi-stage purposive sampling to select the district and two HIV Care and Treatment Centers (CTCs), then randomly sampled participants from a pool of 4,000 adults PLH registered and attending HIV care and treatment services at the two CTCs. Due to logistical limitations, we were not able to implement the study in all CTCs in Dar es Salaam city, so we used a sampling approach to obtain the most relevant and representative sample. Purposive sampling was used to first select a larger district, Kinondoni, out of the three districts in Dar es Salaam region. The same approach was used to select two larger CTCs within the Kinondoni district (two CTCs were selected that had enrolled at least 2,000 patients receiving ART before the study started) to ensure wider representation of the population. Based on a target sample size of 320 and an estimated 20% loss to follow-up, we randomly selected 400 PLH to participate in the study. Every third person arriving at the study clinics from January to August 2012 was selected to determine eligibility. If he/she was eligible, then the prospective study participant was asked to be part of the study through informed consent. Among those eligible, participated in the study. If the selected participant was eligible but not willing to participate in the study RAs used the same approach to randomly select another participant until the sample of 400 was reached at baseline. Our analysis was restricted to 326 (84.5% of the sample) participants who had data at both baseline and 24 months of follow-up and had attended at least one *NAMWEZA* session in order to ensure they were exposed to the intervention.

For the qualitative data, a purposive sampling approach was used to select study participants that had attended more than half (at least 6 sessions) of the *NAMWEZA* training to ensure they had adequately experienced the intervention to provide detailed responses to the targeted study questions. Five informants were selected from each of the four age and gender groups making a total of 20 participants.

The eligibility criteria included: being HIV-positive; 18 years of age or above; living in the catchment area of the study CTCs; registered for HIV care and treatment at the selected CTCs for at least three months; planning to live in the area for at least two years; and able and willing to provide informed consent. Participants having health problems that hindered attending the *NAMWEZA* training sessions or with plans to leave the district precluding them from participating in 24 months of follow-up were excluded.

### 2.3. Data collection tools and approach

Quantitative interviews were conducted using Audio Computer-Assisted Self-Interview (ACASI) software. The ACASI questions included sociodemographic characteristics, HIV knowledge, communication with their at-risk NMs about HIV prevention, hope, stigma, social support, and self-efficacy for condom use and disclosure. A trained research assistant was available to support the participants by orienting them on ACASI and allowing them time to fill a trial ACASI version to ensure they were comfortable to use ACASI before starting the survey questions. The RAs remained at a separate room where the participant could contact them for any technical help during the ACASI interviews. In addition, a paper-based tool on condom use and abstinence was administered only at follow-up at 24 months to get a general view of participants’ experiences.

For the qualitative data, in-depth interviews were conducted using a semi-structured interview guide with open-ended questions that included probes to explore themes. The tool was developed by the authors, translated and back-translated from English to Swahili, piloted among PLH (not based at the study sites), and revised before being used to collect the study data.

#### 2.3.1. Quantitative measures

We measured three variables of HIV communication as our primary outcome measures for the analysis. These variables were treated as continuous variables and included specific communication about condom use or abstinence, HIV testing and protecting others from HIV. The participants were asked how many times they communicated/discussed with their NMs about the three communication variables in the past three months. The questions included; question 1: Within the past three months, how many times did you communicate/discuss/with your NMs about condom use? Question 2: Within the past three months how many times did you communicate/discuss with your NMs about HIV testing? question 3: Within the past three months how many times did you communicate/discuss with your NMs about protecting others from HIV? The responses to all the three questions were ordinal ranging from the smallest score of 1 to the highest of 4. These included (1) Not at all (never communicated), (2) A few times (1–2 times), (3) Sometimes (3–14 times), (4) Often (15 times and more)

The predictors of communication were measured using structured scales with cross-cultural resonance for the following: the 13-item Self-efficacy for Safe Sex scale (Asante & Doku, 2010; Primdahl, Wagner, & Hørslev-Petersen, 2010) with response options ranging from 1 = “not at all confident” to 4 = “very confident;” the Perceived HIV stigma scale (Berger, Ferrans, & Lashley, 2001; USAID, 2005) with responses ranging from 1 = “ disagree strongly” to 5 = “agree strongly”. Based on the way the questions were framed, stigma responses were reverse coded to indicate more stigma with a score = 1 and less stigma for a score = 5. The HIV knowledge assessment was derived from the Tanzanian Demographic and Health Survey (DHS) phase six assessment 2013 (TACAIDS 2012) including 12 items that showed 67% correct responses and was categorized in analyses as low (0%-33%) moderate (34%-60%) and higher knowledge (61%-100%); and the 10-item Duke University-UNC Functional Social Support Questionnaire (Broadhead, Gehlbach, De Gruy, & Kaplan, 1988) for social support with response items ranging from 1 = “as much as I would like” and 4 = “never.” Items for this scale were reverse coded in this study to indicate greater social support for a score of 4 and lower for a score of 1. Other predictors included the number of *NAMWEZA* sessions attended as a measure of the dose of exposure to the intervention. Other covariates and potential confounding variables that were measured included age, gender, marital status, employment status, education levels and wealth index which were measured and analyzed as binary or ordinal variables. The wealth index was computed based on a list of items reflecting socioeconomic status that were collected during the baseline interviews using the Filmer and Pritchett approach (Filmer & Pritchett, 2001). General feedback indicators included the type of NM communicated with, how hard or easy it was to share HIV prevention messages, their experiences communicating with spouses/sexual partners and what helped to facilitate communication.

#### 2.3.2. Qualitative assessment

The aim of this section was to obtain from the participants the depth explanations on their overall experiences of communicating HIV prevention with NMs using the *NAMWEZA* strategies and to explore findings in the quantitative section. The semi-structured in-depth interview guide explored two thematic areas including I) experiences in communicating HIV prevention with NMs after the training; ii) strategies used to communicate with some types of NMs including spouses and children who from the quantitative section only about 29% of participants reported to have communicated with (Table 2). How they used *NAMWEZA* training skills to avoid HIV related stigma which from the quantitative section appeared to hinder communication (Table 4). ii) How they initiated and sustain such communications with specific probes for *NAMWEZA* communication strategies and techniques for talking about HIV prevention. Information was summarized on types of NMs, communication strategies applied that worked well or not, challenges faced and related experiences that PLH were willing to share.

### 2.4. Data management and analyses

The quantitative data from ACASI was cleaned by the research team data clerk at the end of the day by checking for missing or incomplete data and feedback was provided to the field RAs to minimize systematic errors. The clean data set was exported to SPSS version 20 for analysis using Stat/Transfer software. Descriptive analyses included means and standard deviations (SDs) to summarize continuous data and frequencies for categorical variables. Chi-square tests were used to examine differences in the proportions of responses to the feedback indicators across the four study groups. To assess changes in communication variables from baseline to 24 months of follow-up after the *NAMWEZA* intervention, t-tests for paired data were performed for each communication variable.

To identify factors associated with HIV prevention communication with NMs we performed bivariate and multivariate ordinal logistic regression analyses. We started with bivariate models for each predictor variable and the three communication outcome variables. This was followed by multivariate logistic regression including only the predictor variables that had a p-value of less than 0.2 in the bivariate analyses. The multivariate models included predictor variables such as the number of *NAMWEZA* training sessions attended (dose of *NAMWEZA* exposure), HIV knowledge scores, self-efficacy for condom use and abstinence, stigma, and social support. Other variables included gender, age, marital status, education level, employment, wealth index to control for confounding. This approach was repeated for each of the three communication variables: communication about condom use and abstinence; communication about HIV testing; and communication about protecting others from HIV.

Qualitative data were analyzed iteratively; audio-recorded narratives and data in the form of field notes were transcribed by social scientists and reviewed on a weekly basis to ensure that any new information was identified by the study team and included for probing in subsequent in-depth interviews. This iterative approach was used in order to maximize saturation and also ensure quality data collection. Codes and their definitions were developed as a team after reading at least half of the transcripts and a common codebook was developed that was refined after a validation process at the end of data collection. Data were uploaded into NVivo 8 software and coded, then analyzed based on grounded theory (Wainwright, 1994). The interpretation involved the development of topic codes, categories and finally emergent thematic areas which are illustrated with relevant quotes.

### 2.5. Ethical considerations

Participation in this study was voluntary and only those who provided written informed consent participated. The study was reviewed and approved by the institutional review board (IRB) of the National Institute for Medical Research (NIMR) in Tanzania.

## 3. Results

### 3.1. Sociodemographic characteristics and baseline measures of the study participants

Table 1 displays the overall and group-specific percentages and means distributions of the study participants’ sociodemographic characteristics, study outcome variables, and predictors of interest at baseline. The majority (59%) of participants were women, 53% were living with a sexual partner, and 35% were self-employed. Over half (56%) of the participants fell within the moderate wealth index category and about 53% had low HIV knowledge. The average age of the study participants was 39 years. The mean scores on communication with NMs about HIV prevention for all three communication variables were fairly low, with average scores of 2.0 (SD = 0.6), 2.0 (SD = 0.7), and 1.0 (SD = 2.0) for condom use/abstinence, uptake of HIV testing, and protecting others from HIV, respectively. See Table 1 for more details on the baseline measures and variations across the four study groups.

### 3.2. Overall feedback from the participants at the end of 24 months follow up

Table 2 shows the percentage of NMs that the PLH frequently communicated with and feedback on how difficult or easy it was to communicate about HIV prevention after completing the training and at the end of 24 months of follow-up. The overall average number of *NAMWEZA* sessions attended by participants was high: 7.7 (SD = 3.4) which is above half of the expected 10 sessions. It was easier for PLH to communicate about HIV prevention with their friends compared with their spouses and children (p < 0.001). More men reported that speaking with their sexual partners about HIV prevention was very easy compared to women, for both younger (46% for men versus 25% for women) and older groups (38% for men versus 10% for women). These differences were statistically significant (p < 0.001). In addition, the respondents pointed to factors or characteristics in both themselves and their NMs that made it easier to hold a conversation/discussion about HIV prevention—these included the level of NMs’ awareness/knowledge of HIV, NMs being HIV-positive, and the respondent having disclosed their HIV status to their NMs sometimes in the past (p < 0.001).

### 3.3. Changes in communication from baseline to 24 months of follow-up

From baseline and 24 months follow up of the *NAMWEZA* intervention there was a significant increase in the mean scores of communication about condom use and/or abstinence by 0.28 (SD = 0.14), p = 0.012, and the mean scores of communication about HIV testing increased by 0.42 (SD = 0.11), p < 0.001, while the mean scores of communications about protecting others from HIV did not change significantly; mean was 0.01(SD = 0.005), p = 0.890. These results were obtained by paired t-tests and are not presented in the tables.

### 3.4. Predictors of communication at the end of 24 months of follow up

Tables 3, 4 and 5 show bivariate and multivariate predictors of each of the three communication variables. Table 3 shows multivariable models predicting communication about condom use and abstinence. In the bivariate model (model 1) being single as opposed to married, older than 35 years of age, the number of sessions attended, and self-efficacy predicted communication (p < 0.05). In the multivariate analysis (model 2) the same variables predicted communication about condom use and abstinence were significant (p < 0.05), with the exception of stigma which indicated a negative association meaning the participants with high stigma communicated less.

Table 4 shows the predictors of frequency of communication about the uptake of HIV testing. In the bivariate model (model 1) marital status, age, HIV knowledge, number of *NAMWEZA* sessions attended, stigma and self-efficacy were significant predictors (p < 0.05). In the multivariate model (model 2) the same variables were all significant predictors (P < 0.05). Table 5 shows the predictors of communication about protecting others from HIV. Only female gender and age (older than 35 years as opposed to younger) significantly predicted the frequency of communicating with NMs (p < 0.05) in both the univariate and multivariate analyses.

### 3.5. Qualitative results

Themes from the qualitative data included the impact of gained knowledge on communication, using specific *NAMWEZA* communication skills, and facilitators as well as challenges of communicating HIV prevention as well as specific strategies used to communicate to spouses, children and deal with stigma as experienced by the participants

#### 3.5.1. Gaining new HIV knowledge and skills

PLH described overcoming obstacles to communicating HIV prevention after completing *NAMWEZA* as summarized in the following quote:

“…After the NAMWEZA it became easier to talk to them [his co-workers] about condom use, having one partner and so forth. I could not overcome before [before NAMWEZA] because I knew little and I didn’t know. I made them [co-workers] think more and see the reality of the negative consequences if they will not prevent themselves from HIV” (Older man, 53 years).

“I got more information on issues I misunderstood prior to attending NAMWEZA training… I even asked the facilitator more questions at the end of the sessions just to be sure about this and other issues which I wasn’t sure of concerning HIV prevention behaviors. For example I used to think that once both sexual partners are infected there is no any danger of new infections so they don’t need condoms but I learned in NAMWEZA that an HIV infected person can get re-infected with another type of HIV virus from an HIV infected sexual partner which can sometimes be more difficult to treat, so I see the importance of condom use even if your partner is also HIV positive.…” (Younger Man, 33 years).

Another participant mentioned “I don’t have that much education and I fear I might say the wrong things and they will laugh at me, but now [after NAMWEZA] I have taught a lot about HIV prevention; I am comfortable when I talk nowadays” (Older woman, 60 years).

#### 3.5.2. Experiences using specific NAMWEZA communication skills

Participants used a range of *NAMWEZA* based communication skills for HIV prevention with NMs including circular questioning, “I”- statements, ability spotting, using verbal and non-verbal communication skills and valuing others more through viewing them more positively as summarized in the following quote: “I begin discussions by making others (NMs) feel better about themselves that includes my husband . For example, I pick what is good about them (NMs) and tell them before start by telling about HIV prevention. You know like my husband is a polite person and a good father because he treats the children so well. He becomes happy when I tell him that he is a good father…you see what I mean.? they feel valued! It (the approach of spotting abilities and valuing people in your conversation) makes people want to know more and prevent themselves from HIV because they start seeing that they are of value” (Younger woman, 22 years).

“For example, the session on assertiveness…and made me able to use this assertive approach to probe and make them [NMs] see the reality and consequences of not preventing themselves from HIV even though they don’t know my HIV status” (Older man, 53 years).

#### 3.5.3. Other facilitators and hindrances to successful discussions of HIV prevention with NMs

Participants also described their experiences in communicating about HIV prevention with their NMs as easier when done among peers in the following quotation: “For friends, it’s easier for me to open up to discuss HIV with than my family members (respondent and his friends) because these are our issues” (Younger man, 19 years old).

Others described communication was easier if both spouses were HIV positive: “With my husband, it’s very easy to discuss HIV prevention, like we talk about condoms because we are both HIV-positive and we want to remain healthy” (Older woman, 62 years).

Specific strategies to communicate HIV prevention with difficult types of NMs.

The quantitative section (Table 1) indicated that only 29% of the study participant did report communicating with children or spouses. Likewise, stigma was a negative predictor of communication. In the qualitative section participants seem to acknowledge that it is culturally hard especially for children and they mentioned some of the skills from *NAMWEZA* they used to address that cultural hindrances as narrated in the following quotation, “Using newly learned verbal and non-verbal communication skills, including listening for positive feedback opportunities, was also used to minimize chances of arousing anger and to assertively communicate with NMs as illustrated in this quotation, “I learned from NAMWEZA the way to have the upper hand in any discussion. It works now I can speak to my children about HIV prevention you know that is hard in our culture even to mention the word condom to your teenager, but through skills, I got from NAMWEZA. I mean .. through eye contact, voice tone and the way I hold my posture, and avoiding arousing anger, I give positive feedback, not just negative, they (his children) like talking to me nowadays compared to the past when they used to avoid me” (Older man, 48 years).

“My husband has stopped coming late at night and drinking (alcohol) because I used the I statement which I learned from NAMWEZA. I just say to him… I (her) will be happier if you(her husband) came earlier instead of the verbal fight I used to put up with him before and he listens more even last week he didn’t know his HIV status through this method I was able to make him visit a testing center” (young women 28 years)

Another hindrance was participant described how she communicates HIV prevention to her friends without the friends realizing her HIV positive status as a way of avoiding stigma in the following quotation, “I work in a bar and I have so many friends, because I know a lot about HIV and how its transmitted (since I have been HIV positive for 9 years) I used to keep quiet and not tell them…because of the shame and everyone in the bar will point a finger at me that you must be HIV infected that is why you know much but this has changed, after *NAMWEZA* I always use circular questioning…ha.haaa! (lough) see because it makes a person see logical things and answer without ever pointing to your HIV status” (Older women 41 years)

## 4. Discussion

We found that the mean communication scores about condom use and abstinence and HIV testing uptake significantly increased at the end of 24 months, while communication about protecting others from HIV did not change. Higher levels of HIV-related knowledge, self-efficacy for safe sex and disclosure, older age, a higher number of *NAMWEZA* sessions attended, and being female were important predictors of increased frequency of HIV prevention communications between PLH and their NMs.

These quantitative findings are supported by the narratives of participants’ experiences with such communications, which largely depicted the roles of new knowledge and skills gained from *NAMWEZA* in removing existing individual concerns and fears and building self-confidence and motivation for PLH to initiate HIV prevention discussions with their NMs who are most at risk of acquiring HIV infection. The results also demonstrated the effects of age and gender on HIV prevention communications in this population, which has been reported from previous studies (Anugwom & Anugwom, 2016; Peltzer & Matseke, 2013).

However, in this study, the female gender positively predicted communications about protecting others from HIV unlike other studies (Albarracin, Kumkale et al. 2004; Exavery et al., 2012; McCloskey et al., 2005; Nyamhanga & Frumence, 2014; Sa & Larsen, 2008). Previous studies in Tanzania, as in other low income countries where gender inequality exists, show that women are limited in communicating freely about HIV prevention or engaging in cross-gender dialogue about safe sex or testing for HIV (Albarracin, Kumkale et al. 2004; McCloskey et al., 2005; Nyamhanga & Frumence, 2014; Sa & Larsen, 2008). Findings from the current study suggest that *NAMWEZA* potentially addressed what is traditionally expected of women in terms of limiting HIV prevention communication.

Older age was positively associated with all communication variables while the younger men and women reported feeling more comfortable talking to peers about HIV transmission. This could indicate that although cultural norms may still be limiting communication about preventing HIV and other sexually transmitted infections across generations (Albarracin, Kumkale et al. 2004; Asante & Doku, 2010; Exavery et al., 2012; Limaye et al., 2012; McCloskey et al., 2005; Nyamhanga & Frumence, 2014; Sa & Larsen, 2008), with older participants being in a better position to communicate HIV preventive messages across all age groups (Atwood et al., 2012; Kajula et al., 2016; Limaye et al., 2012), the *NAMWEZA* younger participants were comfortable to communicate with peers of similar gender within their NMs. This observation may signify that HIV prevention communication interventions can be embedded in peers of the same gender to avoid the cultural obstacles, similar to the findings of a study exploring attitudes to communicating with NMs about HIV prevention in men who have sex with men (Tobin, Yang, Sun, Pikes, & Latkin, 2014). Talking to sexual partners or children was still reported by the majority of participants to be difficult after completing the intervention. This may be due to the *NAMWEZA* emphasis on PLH communications with peers and friends, rather than with children and spouses, which is supported by the observation that most participants indicated they used *NAMWEZA* strategies to discuss day to day concerns about HIV with peers rather than discussing HIV prevention with their spouses. HIV knowledge and exposure to more sessions was associated with increased preventive communication frequency similar to what other studies have reported (Ross et al., 2007; Rugigana, Birungi, & Nzayirambaho, 2015), indicating that the skills attained by participants could have facilitated communications as has been reported in previous studies (Kattumuri, 2007) (Abramsky et al., 2014; USAIDS, 2012).

This study found that communication about HIV prevention among spouses or sexual partners was facilitated if they were both HIV-positive and if they had disclosed their status to each other. This finding is similar to previous reports of studies that explored spousal communication on sex and HIV-related issues (Konda et al., 2017). Furthermore from the qualitative narratives use of *NAMWEZA* acquired strategies including the “I statements”, ability spotting, posture, voice tones was also used to facilitate more open sexual behavior discussions with NMs specifically among spouses and children. This was important for women since initiating discussions about sex and sexual issues are considered a taboo in the study context (McCloskey & Raphael, 2005; McCloskey et al., 2005; Nyamhanga & Frumence, 2014; Sa & Larsen, 2008).

High HIV knowledge among the participants in these analyses increased the frequency of communications with NM about taking HIV tests. Narrative data indicate that combinations of learned skills for communication and knowledge gained facilitated PLHs’ abilities to communicate HIV preventive messages with their NMs. These findings are similar to previous reports showing that having HIV knowledge only is insufficient to change behavior but embedding skills that help remove perceived individual obstacles might be more helpful (Limaye et al., 2012; Rugigana et al., 2015).

## 5. Study limitations

This study included only participants who completed both baseline follow up interview which is 82% of the 400 participants. This could have a selection bias however the number of those left out is relevantly small and we think the results are still valid and our calculated sample was reached. The study includes qualitative measures which are subjective and difficult to verify but we used the iterative approach of data collection and analysis which allows back and forth feedback between the analysis and data collection.

## 6. Conclusion

We found increased frequency and more successful experiences of interpersonal communication about HIV prevention among PLH and their NMs at risk of acquiring HIV. Although cultural, gender and other individual barriers still existed, the use of *NAMWEZA* specific strategies helped to reduce some of the obstacles to HIV prevention communication. This approach could benefit similar populations where HIV prevention communication is a high priority.

## Figures and Tables

**Table 1.** Baseline characteristics of the study population (N = 326)

Characteristics	Age and gender groups				
	Overall (n = 326) Mean (SD)	Younger men (n = 37) Mean (SD)	Younger women (n = 82) Mean (SD)	Older men (n = 95) Mean (SD)	Older women (n = 112) Mean (SD)
**Age (in years)**	39 (10.0)	30 (4.8)	30 (3.5)	47 (9.1)	43 (7.6)
**Years spent in school**					
Primary school	6 (1.4)	7 (1.3)	7 (1.2)	6.8 (1.0)	6.6 (1.4)
Secondary school	2 (2.6)	2 (1.9)	1 (1.7)	1.1 (2.3)	1.1 (1.7)
Higher education	1 (1.7)	1 (0.2)	0 (0)	0.1 (0.3)	0.14 (0.3)
**Communication about:**					
Condom use/abstinence^1^	2.0 (0.6)	2.0 (0.5)	2.0 (0.6)	2.0 (0.6)	3.0 (0.7)
HIV testing^1^	2.0 (0.7)	2.0 (0.5)	2.0 (0.7)	2.0 (0.6)	2.0 (0.7)
Protecting others from HIV^1^	1.0 (2.0)	1.0 (0.1)	1.0 (0.2)	1.0 (0.2)	1.0 (0.0)
**Hope ^1^**	3.0 (0.7)	2.9 (0.3)	2.4 (0.5)	3.0 (0.5)	2.8 (0.4)
**Self-Efficacy^1^**	2.2 (0.6)	2.1 (0.5)	2.0 (0.6)	2.0 (0.6)	2.5 (0.7)
**Social Support^1^**	2.0 (0.7)	2.1 (0.5)	1.9 (0.7)	2.0 (0.6)	2.3 (0.7)
**Stigma^2^**	1.1 (2.02)	1.1 (0.1)	1.1 (0.2)	1.1 (0.2)	1.1 (0.0)
**Sex**	**n (%)**		-	-	-
Overall Female	**191 (58.5)**				
**Marital status**	**n (%)**	**n (%)**	**n (%)**	**n (%)**	**n (%)**
Living with a partner	173 (53)	17 (46)	46 (56)	27 (28)	70 (63)
Single/widow/widower	152 (47)	20 (54)	36 (44)	68 (72)	42 (38)
**Employment status**					
Employed	44 (13)	3 (8)	15 (18)	12 (13)	14 (13)
Self-employed	113 (35)	11 (27)	20 (24)	46 (48)	36 (32)
Housewife/husband	62 (19)	0 (0)	19 (23)	9 (10)	34 (30)
Unemployed	103 (32)	21 (57)	27 (33)	27 (28)	28 (25)
Student	4 (1)	2 (5)	1 (1)	1 (1)	0 (0)
**Wealth Index’**^5^					
Relatively low	82 (25)	10 (27)	20 (24)	17 (18)	53 (31)
Moderate	182 (56)	23 (62)	44 (54)	59 (62)	56 (50)
Relatively high	62 (19)	4 (11)	18 (22)	19 (20)	21 (19)
**HIV knowledge**					
Low (0–33%)	173 (53.1)	20 (54)	48 (59)	46(49)	59 (53)
Moderate (34–60%)	88 (27.0)	13 (35)	15 (18)	30(32)	30 (27)
High (>60%)	65 (19.9)	4 (11)	19 (23)	30(27)	23 (21)

1 1 = the lowest score, 4 = the highest.

2 1 = highest stigma, 5 = lowest score of stigma.

5 Wealth Index calculated based on Filmer and Pritchett estimation approach (2001).

**Table 2.** Participants responses on communicating HIV prevention with different type of social network members (NMs) at 24 months follow-up (N=326)

	Younger men	Younger women	Older men	Older women	P value
	N=37 n(%)	N=82 n(%)	N=95 n(%)	N=112 n(%)	
**NMs easier to talk with about HIV prevention^1^**					
Spouse/partner/children	7 (19%)	10(12%)	12(13%)	66(59%)	<0.001
Friends	20(54%)	50(61%)	68(72%)	36(32%)	
Other relatives	10(27%)	22(27%)	15(15%)	10(9%)	
**Talking to others on condom use and abstinence^1^**					
Very difficult	5(14%)	4(5%)	8(8%)	31(28%)	
Difficult	4(11%)	7(8%)	8(8%)	21(18%)	<0.0001
Easy	19(53%)	48(59%)	52(55%)	27(24%)	
Very easy	9(22%)	23(28%)	27(29%)	33(30%)	
**Talking to sexual partners about condom use and abstinence ^1^**					
Very Difficult	7(19%)	18(22%)	21(22%)	23(21%)	<0.0001
Difficult	9(24%)	32(39%)	27(29%)	59(54%)	
Easy	5(14%)	12(15%)	11(11%)	20(16%)	
Very easy	16(43%)	20(24)	36(38%)	10(9%)	
**Used NAMWEZA strategies to communicate issues addressing:**					
Daily concerns of HIV transmission	27(73%)	63 (77%)^2^	71 (75%)^2^	51 (46%)^2^	<0.0001
Partner/spouse	10 (27%)	17(21%)	22 (23%)	58 (51%)	
**Others things that made communication easier^1^**					
Network awareness of HIV	19(51%)	48(59%)	51(54%)	36(32%)	
Both HIV infected	3(8%)	11(13%)	5(5%)	36(32%)	<0.0001
Disclosed HIV status	15(41%)	23(28%)	39(41%)	40(36%)	

1 Fishers exact test was used to estimate the P value.

2 Percent not 100 because of missing data

**Table 3.** Bivariate and multivariate analysis for predictors of communication about condom use and abstinence^o^ (N = 326)

Predictors	Model 1*		Model 2**	
	OR (95% CI)	p-value	OR (95% CI)	p-value
Sex^1^	0.910 0.708, 0.784)	0.113	0.936 (0.757, 0.811)	0.365
Marital Status^2^	1.123 (1.040, 1.214)	0.004	1.103 (1.022, 1.189)	0.011
Age^3^	1.242(1.071, 1.441)	0.004	1.217 (1.054, 1.404)	0.007
Wealth Index	1.084(1.036, 1.218)	0.170	1.064 (1.052, 1.191)	0.280
Education Levels^4^	1.053 (1.038, 1.123)	0.175	1.061 (1.057, 1.101)	0.277
HIV Knowledge	1.066(1.005, 1.004)	0.011	1.049 (1.007, 1.063)	.021
Sessions Attended	1.092(1.012, 1.045)	0.0001	1.082 (1.019, 1.064)	.000
HIV Stigma^5^	0.742(0.559, 0.984)	0.039	0.951 (0.865, 0.899)	0.074
Self-efficacy for safe sex and disclosure^6^	1.477 (1.296, 1.632)	0.001	1.438 (1.254, 1.649)	0.000
Social Support^7^	1.066 (1.049, 1.079)	0.742	1.004 (1.049, 1.058)	0.874

0 Condom use and abstinence were asked as one question and measured as one variable.

* Bivariate model,

** Multivariate model controlling for social demographic factors.

1 1 = Male, 2 = Female,

2 1 = Living with a partner 2 = Single/widow/widower,

3 Age group 1 = 18–30 years, Age group 2 ≤ 30 years.

4 1 = Less than primary, 2 = Completed primary, 3 = Secondary education, 4 = Higher education (university and college).

5 1 = highest stigma, 5 = lowest score of stigma,

6 1 = Low self-efficacy for sex and disclosure, 4 = high self-efficacy for safer sex and disclosure

7 1 = low social support, 4 = high social support

**Table 4.** Bivariate and multivariate analysis of determinants of communication about HIV testing (N = 326)

Predictors	Model 1*		Model 2**	
	OR (95% CI)	p-value	OR (95% CI)	p-value
Sex^1^	0.916 (0.878, 0.946)	0.227	0.917 0.882, 0.950)	0.216
Marital status^2^	1.150 (1.068, 1.240)	0.000	1.117 (1.039, 1.202)	0.003
Age^3^	1.252 (1.088, 1.442)	0.002	1.229 (1.069, 1.413)	0.004
Wealth index	1.103 (1.065, 1.115)	0.082	1.095 (1.017, 1.221)	0.098
Education^4^	1.043 (1.029, 1.097)	0.496	1.003 (1.001, 1.011)	0.987
Knowledge of HIV	1.040 (1.025, 1.059)	0.022	1.030 (1.003, 1.059)	0.029
Session participation	1.051 (1.014, 1.087)	0.004	1.043 (1.014, 1.071)	0.004
HIV related Stigma^5^	1.097 0.887, 0.979)	0.005	1.087 (1.155, 1.021)	0.008
Self-efficacy for sex and disclosure^6^	1.051 (1.131, 1.405)	0.0001	1.267 (1.108, 1.391)	0.001
Social support^7^	1.051 (1.048, 1.083	0.904	1.003 (1.048, 1.054	0.904

* Bivariate model,

** Multivariate model controlling for sociodemographic confounders.

1 1 = Male, 2 = Female,

2 1 = Living with a partner 2 = Single/widow/widower,

3 Age group 1 = 18–30 years, Age group 2 ≤ 30 years.

4 1 = Less than primary, 2 = completed primary, 3 = secondary education, 4 = high education (university and college), 5 = lowest score of stigma,

6 1 = Low self-efficacy for sex and disclosure,4 = high self-efficacy for safer sex and disclosure

**Table 5.** Bivariate and multivariate analysis, predictors of communication about protecting others from HIV (N = 326)

Predictors	Model 1*		Model 2**	
	R (95% CI)	p-value	R (95% CI)	p-value
Sex^1^	1.153 (1.021, 1.177)	0.021	1.153 (1.014, 1.196)	0.029
Marital Status^2^	1.042 (0.8890.975,)	0.492	0.980 (0.865, 0.984,)	0.548
Age^3^	1.150(1.018, 1.177)	0.024	1.145 (1.011, 1.220)	0.033
Wealth Index	1.036 (1.026, 1.145)	0.496	1.031 (1.021, 0.142)	0.552
Education Levels^4^	1.009 (1.004, 1.021)	0.902	1.005 (1.003, 1.019)	0.949
Employment	1.115 (1.096, 1.132)	0.107	1.106 (1.023, 1.149)	0.144
HIV Knowledge	1.051 (1.498, 1.105)	0.094	1.143 (1.127, 1.178)	0.874
Sessions Attended	1.010 (1.002, 1.015)	0.311	1.009 (1.006, 1.026)	0.325
HIV Stigma^5^	0.985 (0.935, 0.989,)	0.600	0.985 (0.954,−0.990)	0.642
Self-efficacy for safe sex and disclosure^6^	1.042 (1.014, 1.087)	0.401	1.035 (1.024, 1.108)	0.495

* Bivariate model,

** Multivariate model controlling for sociodemographic confounders.

1 1 = Male, 2 = Female,

2 1 = Living with a partner 2 = Single/widow/widower,

3 Age group 1 = 18–30 years, Age group 2 = >30 years.

4 1 = Less than primary, 2 = Completed primary, 3 = Secondary education, 4 = Higher education (university and college),

5 1 = highest stigma, 5 = lowest score of stigma,

6 1 = Low self-efficacy for sex and disclosure, 4 = high self-efficacy for safer sex and disclosure

## PUBLIC INTEREST STATEMENT

Interpersonal communication to prevent HIV transmission between people living with HIV (PLH) and their social network members increased after receiving the *NAMWEZA* (yes I/we can) intervention. PLH were taught how to become more positive by looking at good from another person instead of blames then communicate positively to invite discussions without arousing anger.

Although the participants faced challenges talking to children, spouse and stigma, but the PLH were able to use some of *NAMWEZA* approaches including *ability spotting, “I statement” and circular questioning etc* to bypass these obstacles. This approach can be used in similar places to improve the capacity of PLH to communicate with people at risk of HIV about HIV prevention.

The intervention didn’t improve communication about protecting others from HIV. This could be partly due to the stigma which is still high in Tanzania or some other reasons which can be explored through future researches.
